# A 2015 outbreak of Getah virus infection occurring among Japanese racehorses sequentially to an outbreak in 2014 at the same site

**DOI:** 10.1186/s12917-016-0741-5

**Published:** 2016-06-10

**Authors:** Hiroshi Bannai, Akihiro Ochi, Manabu Nemoto, Koji Tsujimura, Takashi Yamanaka, Takashi Kondo

**Affiliations:** Equine Research Institute, Japan Racing Association, 1400-4 Shiba, Shimotsuke, Tochigi 329-0412 Japan

**Keywords:** Getah virus, Japan, Racehorses, Sequential outbreak

## Abstract

**Background:**

As we reported previously, Getah virus infection occurred in horses at the Miho training center of the Japan Racing Association in 2014. This was the first outbreak after a 31-year absence in Japan. Here, we report a recurrent outbreak of Getah virus infection in 2015, sequential to the 2014 one at the same site, and we summarize its epizootiological aspects to estimate the risk of further outbreaks in upcoming years.

**Results:**

The outbreak occurred from mid-August to late October 2015, affecting 30 racehorses with a prevalence of 1.5 % of the whole population (1992 horses). Twenty-seven (90.0 %) of the 30 affected horses were 2-year-olds, and the prevalence in 2-year-olds (27/613 [4.4 %]) was significantly higher than that in horses aged 3 years or older (3/1379 [0.2 %], *P* < 0.01). Therefore, the horses newly introduced from other areas at this age were susceptible, whereas most horses aged 3 years or older, which had experienced the previous outbreak in 2014, were resistant. Among the 2-year-olds, the prevalence in horses that had been vaccinated once (10/45 [22.2 %]) was significantly higher than that in horses vaccinated twice or more (17/568 [3.0 %], *P* < 0.01). Horse anti-sera raised against an isolate in 2014 neutralized both the homologous strain and a 2015 isolate at almost the same titers (256 to 512), suggesting that these viruses were antigenically similar. Among horses entering the training center from private surrounding farms in 2015, the seropositivity rate to Getah virus increased gradually (11.8 % in August, 21.7 % in September, and 34.9 % in October). Thus, increased virus exposure due to the regional epizootic probably allowed the virus to spread in the center, similarly to the outbreak in 2014.

**Conclusions:**

The 2015 outbreak was caused by a virus which was antigenically close to the 2014 isolate, affecting mostly 2-year-old susceptible horses under epizootiological circumstances similar to those in 2014. The existence of 2-year-olds introduced from regions free from Getah virus could continue to pose a potential risk of additional outbreaks in upcoming years. Vaccination on private farms and breeding farms would help to minimize the risk of outbreaks.

## Background

Getah virus is classified in the genus *Alphavirus* in the family *Togaviridae* [1]. It is mosquito borne and is widespread from Eurasia to Australasia. This virus causes fever, generalized rash, and edema of the legs in horses [1], and it causes fetal death and reproduction disorders in pigs [2, 3].

We previously reported that an outbreak of Getah virus infection occurred in racehorses at the Miho training center of the Japan Racing Association in autumn 2014, affecting 33 horses [4]. It was the first reported outbreak of infection with this virus among vaccinated horse populations worldwide, and the first one in Japan since 1983 [5]. The indirect causes of this outbreak included the existence of susceptible horses that did not complete the vaccination program at the training center and an increased risk of exposure because of epizootic infection around the training center [6]. However, the direct cause of the outbreak was still unclear, and the epizootic pattern of this re-emerging virus in upcoming years was unpredictable.

Following the outbreak in 2014, we took control measures to prevent a possible outbreak in the coming season. Measures included the reinforcement of pest control for vector mosquitoes at the training center and recommendations for Getah virus vaccination and pest control on the private farms surrounding the center. However, in 2015, another outbreak of Getah virus infection occurred at the Miho training center—the same site as in 2014. Here, we summarize the epizootiological aspects of the current outbreak and analyze the antigenic properties of the isolated virus to estimate the risk of possible outbreaks in upcoming years.

## Methods

### Study site

The Miho training center is in Ibaraki Prefecture in the Kanto region of Japan. About 2000 racehorses are trained at the center, and about 1000 racehorses are replaced with new ones every month. The horses are generally accommodated at the center for 1 to 6 months for training. After they leave the center they are usually kept on other farms for several months for rest; they then re-enter the center. The Japan Racing Association is the sporting authority which administers the horses in the training center, and the clinical samples were collected as a part of regular activities for disease prevention. The owners of racehorses have been notified that their horses might be subjected to mandatory sampling of clinical specimens for diagnostic and research purposes.

A two-dose priming course of Getah virus vaccine is given to 2-year-olds. The vaccination period generally starts in May and finishes in October to cover the mosquito season. Horses that are present at the training center in spring receive the first dose in May and the second dose in June. In the case of horses that enter after the mosquito season has started, the first dose is administered when they enter, and the second dose is given about 1 month after the first. From the second season onward, the horses are vaccinated annually before mosquito season.

### Prevalence of Getah virus infection among populations stratified by age and number of vaccine doses received

We investigated the age distribution and vaccination histories of horses that were present at the Miho training center on August 15, 2015, i.e. a few days before the outbreak started (*n* = 1992). On the basis of the number of vaccination doses they had received before the outbreak, horses in populations of each age were categorized into two groups, namely 1) one dose; and 2) two doses or more. The prevalence of disease onset of Getah virus infection in each population during the period from August 15 to October 30 2015 was calculated by dividing the number of affected horses in each category by the number of horses in the corresponding population. The statistical significance of differences in prevalence was evaluated by using Fisher’s exact test.

### Cell culture

For virus isolation and virus-neutralizing (VN) testing, we used Vero cells (Sumitomo Dainippon Pharma, Tokyo, Japan). Cells were cultured in minimum essential medium (MEM, MP Biomedicals, Irvine, CA, USA) containing 10 % fetal calf serum (Sigma Aldrich Inc., St. Louis, MO, USA), 100 units/ml penicillin, and 100 μg/ml streptomycin (Sigma Aldrich Inc.). MEM containing 2 % fetal calf serum, 100 units/ml penicillin, and 100 μg/ml streptomycin was used as a maintenance medium for virus isolation and VN testing.

### Detection of Getah virus RNA in blood samples of pyretic horses at the Miho training center in 2015

Viral gene detection was performed in blood samples of pyretic horses (≥38.5 °C) at the Miho training center. The test period started in June 2015 and finished in mid-November 2015. Viral RNA was extracted from EDTA-treated blood samples by using a nucleic acid isolation kit (MagNA Pure LC Total Nucleic Acid Isolation Kit, Roche Diagnostics, Mannheim, Germany), and viral gene detection was performed by an RT-PCR for the Getah virus *nsP1* gene using primer sets M_2_W-S and M_3_W-S [7]. For some of the positive samples, the RT-PCR products (*n* = 7) were sequenced as described previously [4].

### VN test for Getah virus in paired sera collected from pyretic horses at the Miho training center in 2015

From among 95 horses that developed pyrexia between 1 August and 30 October at the Miho training center, we collected acute and convalescent sera (2- to 11-week intervals between paired sera collection) from 52. These included 14 horses that were positive and 38 that were negative on the above-mentioned Getah virus RT-PCR. The sera were subjected to a VN test for Getah virus using the 14-I-605 strain, which had been isolated from a racehorse during the 2014 outbreak, as described previously [6]. The VN test for Getah virus was performed as described previously [6]. The VN titer was defined as the reciprocal of the highest dilution that completely inhibited virus growth. Horses that showed a ≥4-fold increase between the paired sera were defined as seroconverted.

### Virus isolation

EDTA-treated blood samples collected from 23 horses that had developed pyrexia and were positive on Getah virus RT-PCR during the period from 18 August to 26 September 2015 were used for virus isolation. For some of the 23 horses, buffy-coat specimens containing leukocytes instead of whole blood were used for virus isolation. Getah virus was isolated by using Vero cells as described previously [6]. Briefly, the samples were frozen and thawed three times and then centrifuged at 800 *g* for 20 min at 4 °C. The supernatants were inoculated onto 1-day monolayer cultures of Vero cells or inoculated with the Vero cells simultaneously. The next day, the cells were washed three times with phosphate-buffered saline (pH 7.2) and cultured in maintenance medium. To identify Getah virus–specific nucleotide sequences, the supernatants of specimens that showed cytopathic effects were tested by RT-PCR for the *nsP1* gene, as described above.

### Antigenic comparison of the vaccine strain and Getah virus strains isolated in 2014 and 2015

Cross-neutralizing tests between the strain isolated in 2014 (14-I-605), the strain isolated in 2015, and the vaccine strain (MI-110) were performed. Horse anti-sera that were raised against the MI-110 strain (*n* = 2) and 14-I-605 strain (*n* = 2) and prepared in our previous study were used [8]. VN tests were performed as described above.

### Investigation of Getah virus epizootic infection among horses on surrounding farms in Ibaraki and Chiba prefectures

Among horses that were introduced into the Miho training center between June and October 2015, those that met all of the following criteria (*n* = 51 to 81 in each month) were tested: 1) 2 years old; 2) transferred from a farm in Ibaraki Prefecture or the neighboring Chiba Prefecture; and 3) no history of vaccination with inactivated Getah virus vaccine. Sera collected on the day each horse entered the Miho training center were subjected to VN testing for Getah virus.

## Results

### Detection of Getah virus infection among pyretic horses at the Miho training center in 2015

**Fig. 1:**
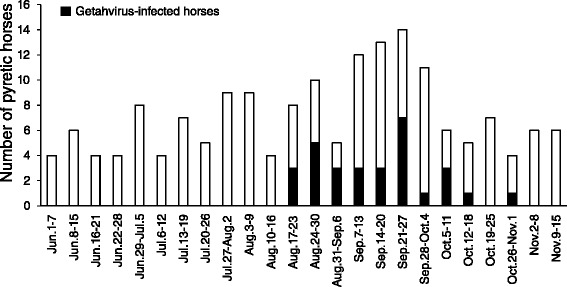
Numbers of pyretic horses and Getah virus–infected horses at the Miho training center. Bars indicated the number of pyretic horses each week from 1 June to 15 November. Black, number of horses positive on RT-PCR for Getah virus or seroconverted to Getah virus on VN testing, or both

The numbers of pyretic horses each week at the Miho training center are shown in Fig. 1. During the period from June to mid-August, there were 4 to 9 pyretic horses each week. These numbers increased to 10 or more in all of the weeks except one from late August to early October, and thereafter decreased to the earlier baseline. Out of 171 pyretic horses in the whole period, 162 were tested for Getah virus by RT-PCR, and 29 of them were positive. The first and last samples that were positive by RT-PCR were collected from pyretic horses on 18 August and 30 October, respectively (Fig. 1). We collected paired sera from 14 of the 29 RT-PCR-positive horses and subjected them to VN testing for Getah virus; all of them had seroconverted (≥4-fold increase). Paired sera collected from 38 RT-PCR-negative horses that had developed pyrexia between 18 August and 30 October were also tested for VN antibodies; one horse, which had developed pyrexia on 25 August, showed seroconversion. In total, from among the 95 horses that developed pyrexia between 18 August and 30 October, 30 were positive for Getah virus infection by RT-PCR or VN testing, or both (Fig. 1). Among the 30 affected horses, seven (23.3 %) had edema of their legs, and three (10.0 %) had body rashes, but all of them recovered within a few days. Two-year-olds accounted for 27 (90.0 %) of the 30 affected horses; the remainder consisted of two 3-year-olds (two horses) and one 7-year-old. Of the affected 2-year-olds, 10 had been vaccinated only once before disease onset, and 17 had been vaccinated twice. All of the affected horses aged 3 years or older had been vaccinated at least twice.

### Prevalence of Getah virus infection among populations stratified by age and number of vaccine doses received

We investigated the age distribution and vaccination histories of the whole horse population at the Miho training center on August 15, 2015, i.e. a few days before the outbreak started (Table 1). All horses (*n* = 1992) had been vaccinated at least once before the outbreak. Among the 2-year-olds (*n* = 613), 568 (92.7 %) had been vaccinated twice or more, and the remaining 45 (7.3 %) had been vaccinated only once (Table 1). Among the 3-year-olds (*n* = 794), 4-year-olds (*n* =314) and 5-year-olds or older (*n* = 271), almost all horses had been vaccinated two times or more (788 [99.2 %], 312 [99.4 %] and 271 [100.0 %], respectively, Table 1).

**Table 1:** Numbers (%) of horses that had received Getah virus vaccine and were being kept at the Miho training center on August 15 in 2015

	Vaccination dose		Total
Age	One	Two or more	
2	45 (7.3)	568 (92.7)	613
3	6 (0.8)	794 (99.2)	794
4	2 (0.6)	312 (99.4)	314
5 or older	0 (0.0)	271 (100.0)	271
Total	53 (2.7)	1,939 (97.3)	1,992

The prevalence of Getah virus infection in the whole population during the outbreak was 30/1992 (1.5 %). The prevalence in 2-year-olds (27/613 [4.4 %]) was significantly higher than that in horses aged 3 years or older (3/1379 [0.2 %], *P* < 0.01). Among the 2-year-olds, the prevalence in horses that had been vaccinated once was 10/45 (22.2 %); this was significantly higher than that in horses vaccinated twice or more (17/568 [3.0 %], *P* < 0.01).

### Virus isolation and analysis of nucleic acid sequence

We tried to isolate Getah virus from blood samples that were positive on RT-PCR. In testing on 23 blood samples, primary cocultivation resulted in the isolation of two strains confirmed as Getah virus by RT-PCR. We analyzed the sequences of the *nsP1* genes of the two strains and those of the RT-PCR products amplified from some of the clinical samples (*n* = 7). All of the samples analyzed had completely identical nucleic acid sequences, and the sequences were also identical to that of the 14-I-605 strain (381 bases, GenBank/EMBL/DDBJ accession number, LC012885) isolated during the 2014 outbreak [6]. From this result, we used one of the two 2015 isolates (15-I-752) for the further studies.

### Antigenic comparison of the vaccine strain and the 2014 and 2015 Getah virus strains

To assess whether the current Getah virus vaccine was effective against the circulating virus in 2015, we performed cross-neutralization tests between the vaccine strain (MI-110) and the strains isolated in 2014 (14-I-605) and 2015 (15-I-752). The results of the cross-neutralization tests are summarized in Table 2. Our previous report revealed that horse sera (*n* = 2) raised against MI-110 neutralized the homologous virus at titers of 512 and neutralized the 14-I-605 strain at almost the same titers (256) [8]. In the current experiment, the same set of sera neutralized the 15-I-752 strain at titers of 256 (Table 2). The horse sera (*n* = 2) raised against the 14-I-605 strain—which were also used in the previous study [8]—neutralized the homologous virus at a titer of 256 or 512 and neutralized the 15-I-752 strain at titers of 512 (Table 2). These results indicated that the two strains isolated in 2014 and 2015 were antigenically close to each other, and that the current vaccine containing the MI-110 strain was likely sufficiently effective against the circulating viruses.

**Table 2:** Virus-neutralizing titers of sera from horses inoculated with Getah virus MI-110 or 14-I-605 strain

		Strain used in virus-neutralization test		
Inoculated strain	Horse	MI-110	14-I-605	15-I-752
MI-110	1	512^a^	256^a^	256
	2	512^a^	256^a^	256
14-I-605	3	512^a^	256^a^	512
	4	1024^a^	512^a^	512

^a^Data quoted from our previous study [8]

### Investigation of Getah virus epizootic infection among horses on surrounding farms in Ibaraki and Chiba prefectures

**Fig. 2:**
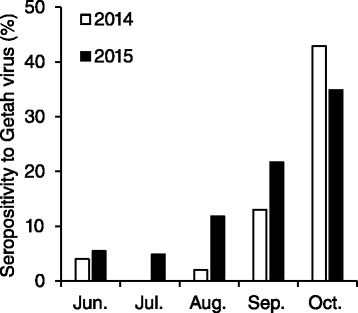
Rates of seropositivity to Getah virus in horses transferred from farms surrounding the Miho training center. Sera were collected from horses introduced to the Miho training center between June to October in 2014 or 2015. The horses (*n* = 51 to 81 in each month) were 2-year-olds that had been transferred from Ibaraki or Chiba prefecture and had no history of Getah virus vaccination. Sera were subjected to VN testing using the 14-I-605 strain. Data for 2014 are quoted from our previous report [6]

During the 2014 outbreak, we found that epizootic Getah virus infection occurred not only at the training center but also on private farms surrounding the center [6]. To assess whether this regional epizootic had also occurred in the 2015 outbreak, we calculated seropositivity rates among horses entering the center from farms in Ibaraki Prefecture and the neighboring Chiba Prefecture each month in 2015. The horses were 2-year-olds with no history of Getah virus vaccination. Seropositivity rates in June (5.5 %) and July (4.9 %) were comparable to those in 2014 (Fig. 2) [6]. An increase in seropositivity was observed in August (11.8 %), and those in September and October were 21.7 and 34.9 %, respectively (Fig. 2), indicating that Getah virus was epizootic in the area in autumn 2015, similarly to the 2014 season.

## Discussion

The epizootic Getah virus infection in 2015 seems to have started earlier than that in 2014, which started in mid-September [4]. This trend was observed both at the Miho training center and on the private farms (Figs. 1 and 2). Despite the early start, the prevalence of Getah virus infection in whole population at the training center in 2015 (30/1992 [1.5 %]) was comparable to that in 2014 (33/1950 [1.7 %]) [4], suggesting that although the epizootic in 2015 started earlier than that in 2014 it progressed more slowly. In support of this finding, the seropositivity rate in horses entering the center was lower in October 2015 (34.9 %) than in October 2014 (42.9 %, Fig. 2). We found that the age proportions of the horses affected in 2014 and 2015 differed greatly; this might have explained the relatively slow mild progression of the 2015 outbreak. The proportion of 2-year-olds among the horses affected in 2014 was 60.6 % [6], whereas that in 2015 was 90.0 %, suggesting that horses aged 3 years or older were relatively resistant to infection. This was probably due to the fact that, because of the 2014 outbreak, many horses aged 3 years or older in 2015 had already been exposed to Getah virus at the center or on the private farms. Therefore, the existence of these resistant horses might have delayed virus spread in the horse population to some extent.

The significantly higher prevalence in the 2-year-olds (4.4 %) than that in the 3-year-olds or older population (0.2 %) was one of the most characteristic aspects of the current outbreak, because such significant difference was not observed in the 2014 outbreak [6]. In this regard, even though older horses at the training center become resistant after natural infection, we are still concerned about the possibility of outbreaks of Getah virus infection in upcoming years. In Japan, more than 95 % of racehorses are bred in Hokkaido Prefecture, in northern Japan, and 2-year-olds are introduced to the training center every year. At the time of introduction, most have not been infected with Getah virus or vaccinated with Getah virus vaccine. Serological surveillance also suggests that there is a low prevalence of Getah virus infection in Hokkaido, with seropositivity rates of 0 % among unvaccinated horses transferred out of Hokkaido in 2013 and 2014 (Bannai et al., unpublished data). Therefore, the existence of newly introduced 2-year-olds could continue to pose a risk of additional outbreaks.

More than 30 private farms are located in Ibaraki Prefecture, where the Miho training center stands, and in neighboring Chiba Prefecture, and horses are repeatedly transferred between the farms and the center for training and rest. Unlike in the training centers, where Getah virus vaccination is mandatory, on the farms vaccination is not common. In addition, this area is one of the biggest producers of pigs. Although the exact prevalence of Getah virus in pigs and its association with the recent outbreaks in horses have not been studied, the horses in this area are considered to be at high risk of infection. As in the 2014 outbreak, in 2015 our results indicated that there was a high prevalence of Getah virus infection among horses on the private farms (Fig. 2). This regional epizootic probably resulted in an increased risk of virus exposure and allowed the virus to spread in the center. Therefore, the 2015 outbreak seems to have occurred under epizootiological circumstances similar to those in 2014. It will be helpful to increase vaccination coverage on private farms to prevent regional virus circulation. Unfortunately, despite our recommendation, coverage on the farms in 2015 seemed to be as low as in previous years, although exact data were not available. In support of this speculation, among the 2-year-olds at the Miho training center in mid-September 2015, the proportion of those that had been vaccinated only once for Getah virus was 9.0 % (75/831 head); this was almost the same as that in 2014 (8.3 % [71/858 head]) [6]. The existence of horses in this category reflects the low vaccination coverage on the private farms, because horses with no history of Getah virus vaccination are given their first, priming, dose on entry to the training center. Among the horses affected in the 2015 outbreak, 33.3 % (10/30 head) had been vaccinated only once before disease onset; this was comparable to the level in 2014 (30.3 % [10/33 head]) [6], suggesting that these horses were highly susceptible and were involved in spread of the virus at the center, similarly to the situation in 2014. The higher prevalence in 2-year-old horses vaccinated only once (22.2 %) than that in those vaccinated twice or more (3.0 %) also suggested the requirement of two-doses priming vaccination for the protection of individual horses. Therefore, further efforts are needed to increase vaccination coverage on the farms.

As described above, the 2014 and 2015 outbreaks of Getah virus infection occurred after an absence of detectable outbreaks for more than three decades. The sudden outbreaks in the two sequential years at the same site suggest the occurrence of direct factors such as mutations that might alter the features of the virus. Although we were initially concerned about antigenic mismatch between the circulating virus and the vaccine strain, our previous and current results suggest that the 2014 and 2015 isolates are antigenically similar to the vaccine strain (Table 2) [8]. Other viral features which may influence the epizootic includes the vector specificity and the efficacy of replication in mosquitoes, because Getah virus is a typical mosquito-borne virus. A previous ecological surveillance in 1979 at the Miho training center revealed that Getah virus was isolated from *Aedes vexans nipponii* and *Culex tritaeniorhynchus*, and these two species were considered to be involved in the circulation of Getah virus in Japan [9]. In our previous study, we compared the full-genome sequences of the isolate in 2014 and the vaccine strain, and found that non-structural protein 3 (nsP3) included 7 amino acid substitutions while the other non-structural proteins had only 1 or 2 substitutions [8]. The carboxyl-terminus domain of nsP3 was reported to be involved in viral replication of genus *Alphaviruses* to which Getah virus belongs [10], and also reported to be a determinant of vector specificity in O’nyoug nyong virus [11]. In this regard, further study on the mutations in the nsP3 protein might provide clues to the causes of the current outbreaks. In addition, investigation of the density of vector species in the region surrounding the training center, the prevalence of Getah virus in the mosquitoes, and the epizootic situation in pigs, the natural host, would help us to understand the risk of future outbreaks in horses.

## Conclusions

In conclusion, an outbreak of Getah virus infection occurred at the Miho training center in 30 horses from mid-August to late October 2015. It was sequential to the 2014 outbreak at the same site. The 2015 outbreak was caused by a virus closely related to the 2014 isolate and affected mostly 2-year-old susceptible horses under epizootiological circumstances similar to those in 2014. The existence of 2-year-olds introduced from non-epizootic regions could continue to pose a risk of additional outbreaks in upcoming years. Vaccination on private farms and breeding farms would help to minimize the risk of outbreaks. Continuous surveillance at the training center, as well on the farms surrounding the center, will be required.

## Abbreviations

MEM, minimum essential medium; nsP3, non-structural protein 3; VN, virus-neutralizing
